# Local iron homeostasis in the breast ductal carcinoma microenvironment

**DOI:** 10.1186/s12885-016-2228-y

**Published:** 2016-03-05

**Authors:** Oriana Marques, Graça Porto, Alexandra Rêma, Fátima Faria, Arnaud Cruz Paula, Maria Gomez-Lazaro, Paula Silva, Berta Martins da Silva, Carlos Lopes

**Affiliations:** Laboratory of Immunogenetics – Autoimmunity and Neurosciences, Unit for Multidisciplinary Biomedical Research (UMIB), Institute of Biomedical Sciences Abel Salazar (ICBAS), University of Porto, Rua Jorge Viterbo Ferreira 228,Edif 2 Piso 4, P-4050313 Porto, Portugal; Pathology and Molecular Immunology Department, Institute of Biomedical Sciences Abel Salazar (ICBAS), University of Porto, Porto, Portugal; Basic and Clinical Research on Iron Biology, Instituto de Biologia Molecular e Celular (IBMC), University of Porto, Porto, Portugal; Instituto de Investigação e Inovação em Saúde (i3S), University of Porto, Porto, Portugal; Hematology Service, Hospital de Santo António, Centro Hospitalar do Porto, Porto, Portugal; Department of Pathology, Portuguese Oncology Institute (IPO), Porto, Portugal; Instituto Nacional de Engenharia Biomédica (INEB), University of Porto, Porto, Portugal; Faculty of Medicine of University of Porto (FMUP), Porto, Portugal; Institute of Molecular Pathology and Immunology, University of Porto, Porto, Portugal

**Keywords:** Breast cancer, Ferroportin 1, Iron, Stromal inflammatory cells, Tissue microenvironment

## Abstract

**Background:**

While the deregulation of iron homeostasis in breast epithelial cells is acknowledged, iron-related alterations in stromal inflammatory cells from the tumor microenvironment have not been explored.

**Methods:**

Immunohistochemistry for hepcidin, ferroportin 1 (FPN1), transferrin receptor 1 (TFR1) and ferritin (FT) was performed in primary breast tissues and axillary lymph nodes in order to dissect the iron-profiles of epithelial cells, lymphocytes and macrophages. Furthermore, breast carcinoma core biopsies frozen in optimum cutting temperature (OCT) compound were subjected to imaging flow cytometry to confirm FPN1 expression in the cell types previously evaluated and determine its cellular localization.

**Results:**

We confirm previous results by showing that breast cancer epithelial cells present an ‘iron-utilization phenotype’ with an increased expression of hepcidin and TFR1, and decreased expression of FT. On the other hand, lymphocytes and macrophages infiltrating primary tumors and from metastized lymph nodes display an ‘iron-donor’ phenotype, with increased expression of FPN1 and FT, concomitant with an activation profile reflected by a higher expression of TFR1 and hepcidin. A higher percentage of breast carcinomas, compared to control mastectomy samples, present iron accumulation in stromal inflammatory cells, suggesting that these cells may constitute an effective tissue iron reservoir. Additionally, not only the deregulated expression of iron-related proteins in epithelial cells, but also on lymphocytes and macrophages, are associated with clinicopathological markers of breast cancer poor prognosis, such as negative hormone receptor status and tumor size.

**Conclusions:**

The present results reinforce the importance of analyzing the tumor microenvironment in breast cancer, extending the contribution of immune cells to local iron homeostasis in the tumor microenvironment context.

## Background

Breast cancer ranks as the most frequent neoplasia and cause of cancer death, in spite of growing advances in early diagnosis and novel therapy regimens [1]. A change in this scenario demands a better understanding of the cellular and molecular processes involved in breast cancer development and progression.

As a fundamental element involved in cell metabolism, division and proliferation, iron has been implicated as an important player in cancer development [2]. The argument that iron may promote the development of breast cancer is supported by animal studies consistently demonstrating that iron-rich diets or iron injected subcutaneously favors breast cancer progression at several stages [3–6]. From the cell biology perspective, it is now well accepted that the malignant state in breast epithelial cells is characterized by a deregulation in cellular iron homeostasis, as revealed by differences in the expression of several iron-related proteins relating with markers of poor outcome [7–11]. Particularly, a marked decrease in the levels of the iron exporter ferroportin 1 is observed both in breast cancer tissue and cancer cell lines with a higher malignancy potential, denoting the relative “iron-deficient” phenotype compatible with their increased proliferative status [12, 13]. In spite of the known impact of genetic and epigenetic changes of breast epithelial cells in tumor progression, it is acknowledged that these are not sufficient for the acquisition of a fully malignant and invasive potential [14–16]. Stromal inflammatory cells, which are present in the breast tissue even before malignant transformation, may also induce alterations in the breast microenvironment that ultimately can drive tumorigenesis [17, 18]. Pioneering studies by De Sousa and co-workers have shown that lymphocytes and macrophages are capable of synthesizing and secreting ferritin [19, 20]. More recent work by Alkhateeb and co-workers not only confirmed that breast cancer-associated macrophages secrete ferritin, particularly in response to pro-inflammatory cytokines, but also that extracellular ferritin stimulates the proliferation of breast cancer cells [21]. Also, Jezequel and co-workers have described ferritin light-chain expression in tumor-associated macrophages with an M2-like phenotype and validated it as a prognostic biomarker in node-negative breast cancer patients [22]. Of note, in vitro M2 polarized macrophages present an iron-release prone phenotype, with higher transferrin receptor 1 and ferroportin 1 expression than classically activated M1 macrophages, which is thought to contribute to its iron recycling function as scavengers of senescent and apoptotic cells and in tissue remodeling [23, 24]. To our knowledge, the expression of ferroportin 1 in breast cancer tumor-associated lymphocytes and macrophages has never been addressed before.

In the present study we analyzed the iron-profiles of epithelial cells, lymphocytes and macrophages in normal human breast and ductal carcinoma samples and assessed their association with clinicopathological markers of cancer progression and behavior. With this approach we reinforce the evidence that favors the contribution of stromal inflammatory cells to breast tumor microenvironment while highlighting the potential role of lymphocytes and macrophages in the regulation of local iron homeostasis.

## Methods

### Sample characterization

Selected and stored human breast tissue samples referred for histological analysis at the Pathology Service at Centro Hospitalar do Porto (between 2004 and 2009), were re-analyzed. We selected 131 samples corresponding to 58 cases of invasive ductal carcinomas (IDC), 16 cases of ductal carcinomas in situ (DCIS) and 57 samples without evidence of breast disease obtained from breast reduction aesthetic surgery, as controls. Axillary lymph node samples from 14 non-metastized and 12 metastized IDC were randomly selected from the initial cohort and analyzed. In addition, 6 frozen core biopsy samples, collected in 2013, from patients with invasive ductal carcinomas from which written informed consent was obtained, were selected for imaging flow cytometry studies. Pathological and clinical features, including histological diagnosis, estrogen receptor (ER), progesterone receptor (PR), human epidermal growth factor 2 (HER-2) status and peripheral white blood cell (WBC) count data were available from the interin pathology reports. ER, PR and HER-2 status were assessed by immunohistochemistry, as routinely done in the Pathology Service. HER-2 ambiguous results were confirmed by FISH.

### Tissue microarray construction

Formalin-fixed paraffin-embedded (FFPE) tissue blocks and hematoxylin and eosin (H&E)-stained slides were retrieved from the archive and re-evaluated by an experienced pathologist (CL). Representative areas from normal breast epithelium, ductal carcinoma in situ and invasive ductal carcinoma lesions were selected, marked on the H&E slides and then sampled into the tissue microarray (TMA) collector blocks. Most selected lesions corresponded to “pure” DCIS or IDC lesions, i.e., from samples with the corresponding classification. Whenever possible, non-malignant and DCIS lesions were also selected from invasive ductal carcinoma cases. Two tissue cores from human donor liver samples were also included in each tissue microarray block, as positive controls. A total of 452 FFPE 2 mm breast tissue cores were used for the tissue microarray construction from which 405 were assessable. 2 μm-thick TMA sections were cut in a microtome and H&E stained. Histologically, each core was classified by the pathologist without previous knowledge of the type of donor sample. Cores with ‘normal’ breast histology retrieved from DCIS or IDC samples were further classified as ‘normal in DCIS’ and ‘normal in IDC’, respectively. Representative areas with malignant lesions from DCIS and IDC were classified as DCIS “pure lesions” or IDC “pure lesions”, respectively. DCIS cores retrieved from IDC samples, without signs of invasion, were classified as DCIS in IDC. The numbers of cores included for each histological type and type of donor sample are summarized in Table 1.

**Table 1:** Number of spots included in TMA receiver blocks

Tissue sample	Type of core	No. of cores in TMA blocks
Control Normal Samples	Normal	119
DCIS	Normal in DCIS	12
	DCIS pure lesion	54
IDC	Normal in IDC	61
	DCIS in IDC	39
	IDC pure lesion	120

*Abbreviations*: *TMA* Tissue Microarray, *DCIS* Ductal Carcinoma *In Situ*, *IDC* Invasive Ductal Carcinoma

### Immunohistochemistry

Immunohistochemical staining was performed in 2 μm-thick TMA sections with the following antibodies: rabbit polyclonal anti-human hepcidin-25 antibody (dilution 1:500, Abcam, Cambridge, UK [25]), rabbit polyclonal anti-human ferroportin 1 antibody (FPN—1:500, Novus Biologicals Europe, Cambridge, UK [26]), rabbit polyclonal anti-human ferritin antibody (FT—1:1000, Sigma-Aldrich, MO, USA [27]), mouse monoclonal anti-human CD71 (TFR1 [clone 10 F11]- 1:80, Novocastra, Newcastle, UK [28]), mouse monoclonal anti-human CD68 (clone Kp-1, 1:2000, A. Menarini Diagnostics, CA, USA), mouse monoclonal anti-human CD163 (clone MRQ-26, 1:100, Cell Marque, CA, USA), mouse monoclonal anti-human CD80 (37711, 1:100, R&D Systems, MN, USA), rabbit polyclonal anti-human CD4 (clone H-370, 1:250, Santa Cruz Biotechnology, TX, USA) and mouse monoclonal anti-human CD8 (clone C8/144B, 1:100, Cell Marque, CA, USA). The sections were deparaffinized twice in xylene, rehydrated in decreasing concentrations of ethanol and washed in water. Heat-mediated antigen target retrieval was done with DakoTarget Retrieval Solution (Agilent Technologies, Denmark). Immunohistochemistry was performed according to Novolink Polymer Detection kit procedures (Leica, Biosystems, Cambridge, UK). Enzyme reactivity was visualized using 3,3′-Diaminobenzidine tetrahydrochloride (DAB, Sigma-Aldrich, MO, USA) and slides were counterstained with Mayers hemalum solution (Merck Millipore, Darmstadt, Germany), dehydrated and mounted with Entellan (Merck Millipore, Darmstadt, Germany). The reaction obtained in all samples was observed in a Leica DM LB microscope. Each antibody optimum dilution was determined in a tissue positive control. Slides with replacement of the primary antibody with an antibody of the same immunoglobulin isotype were integrated in each experiment as negative labeling controls. A section of liver tissue from a HAMP (hepcidin) KO mouse was also included as a hepcidin negative control.

### Staining criteria

Tissue specimens from normal control, DCIS and IDC samples were immunostained for hepcidin, ferroportin (FPN1), transferrin receptor 1 (TFR1) and ferritin (FT) proteins and their cellular localization examined in epithelial cells (Hepcidin, *n* = 323; FPN1, *n* = 315; TFR1, *n* = 308; FT, *n* = 325), lymphocytes (Hepcidin, *n* = 175; FPN1, *n* = 174; TFR1, *n* = 177; FT, *n* = 244) and macrophages (Hepcidin, *n* = 173; FPN1, *n* = 150; TFR1, *n* = 178; FT, *n* = 245). A semi-quantitative evaluation method was applied as follows: the score obtained by the percentage of positive cells (0 % = 0 points; 1–10 % = 1 point:, 11–20 % = 2 points:, 21–35 % = 3 points:, 36–50 % = 4 points: and >50 % = 5 points) was multiplied by the score obtained by the staining intensity (no staining = 0 points, weak staining = 1 point, moderate staining = 2 points and strong staining = 3 points). We are aware that this type of scoring results in a higher number of area groups. However, we considered that grouping the area percentages in groups with higher intervals would also introduce high variation inside each group. Cores from the same donor tissue diagnosed with the same histological type were grouped and their mean score calculated. Lymph node iron-related proteins immunoexpression assessment was done in B cell and T cell areas and in macrophages. Scores ranged from 0 to 3, where 0 was considered absence of immunoexpression, 1, low expression, 2, moderate expression, and 3, high expression of the correspondent iron-related protein.

### Perls’ Prussian blue staining

Hemosiderin deposits were detected by the routine technique of Prussian blue histochemical staining. Briefly, after deparaffinization and rehydration in the ethanol series, sections were immersed in a mixture of equal volumes of potassium ferrocyanide solution and hydrochloric acid solution, both at 2 %. Counterstaining was achieved with nuclear fast red (Merck Millipore, Darmstadt, Germany). The absence or presence of hemosiderin deposits was evaluated in epithelial and stromal inflammatory cells.

### Imaging flow cytometry

OCT-embedded frozen samples from 6 core biopsies were cut in a cryostat and H&E stained for pathological assessment of malign disease. After thawing, biopsies were gently removed with a scalpel and allowed to mechanically disaggregate with the help of forceps. Cells were resuspended in 2 % BSA (Bovine Serum Albumin, Merck Millipore, Darmstadt, Germany) in PBS, and set for staining in a 96-well standard microplate. A Neubauer counting chamber was used in order to count and stain 1 × 10^6^ cells in every assay. After centrifugation at 2000 rpm and resupension in 0.2 % BSA in PBS, cells were incubated with mouse monoclonal anti-human cytokeratin FITC ([clone 1B3] IOTest, Beckman Coulter, Madrid, Spain), mouse monoclonal anti-human CD68 PE-Cy7 ([clone Y1/82A] eBioscience Affymetrix, CA, USA), mouse monoclonal anti-human CD3 PerCP-Cy5.5 ([clone SK7] BD, Madrid, Spain), rabbit polyclonal anti-human FPN PE (Novus Biologicals Europe, Cambridge, UK), mouse monoclonal anti-human CD20 PE-Cy7 ([clone B9E9] IOTest, Beckman Coulter, Madrid, Spain) and FPN PE (Novus Biologicals Europe, Cambridge, UK) [staining 2]. Cells were washed with 0.2 % BSA in PBS and centrifuged at 2000 rpm prior to fixation with Fixation Medium from Fix & Perm Cell Fixation and Permeabilization Kit (Life Technologies, CA, USA) and then resuspended in 0.2 % BSA in PBS for analysis. Single-stained and unstained cells were used as controls. Data were acquired in an imaging flow cytometer (ImageStream®, Amnis, EDM Millipore, Darmstadt, Germany) using a 488 nm laser. Images and data were acquired using INSPIRE Software v4.0 (Amnis, EDM Millipore, Darmstadt, Germany). Brightfield was detected on channel 1, FITC on channel 2, PE on channel 3, PerCP-Cy5.5 on channel 5 and PE-Cy7 on channel 6. A total of 100 μL was loaded per sample and 8000 events meeting the cell classifier were acquired at a 40× magnification (image pixel 0.5 μm^2^). Compensation and analysis were performed in IDEAS v6.0.348 software (Amnis, EDM Millipore, Darmstadt, Germany). Data was compensated through a matrix created based on the single-stained cell controls. A hierarchical gating strategy was created in the software in order to identify breast epithelial cells, lymphocytes and macrophages. Briefly, first focused cells were selected (gradient root mean square of the brightfield) followed by gating on single-cells (brightfield area *vs* aspect ratios). T-Lymphocytes were then selected on an Intensity_CD3 PerCP-Cy5.5 *vs* Area on Channel 1 plot, B Lymphocytes on an Intensity_CD20 PE-Cy7 *vs* Area on Channel 1 plot, macrophages on an Intensity_CD68 PE-Cy7 *vs* Area on Channel 1 plot and finally epithelial cells on an Intensity_cytokeratin FITC *vs* Area on Channel 1. Gated cells were excluded from further analysis before selecting the next population. FPN1 intensity in the cell membrane and cytoplasm was measured through the creation of masks defining the total area of the cell and then the correspondent cytoplasm by eroding the cell membrane in channel 1 (cell membrane = total cell—cytoplasm).

### Laser capture microdissection

Six μm-thick sections from the axillary lymph nodes were cut and placed in PALM® 1.0 polyethylene naphthalate (PEN) membrane slides (Carl Zeiss MicroImaging GmbH, Germany). Before use, slides were treated with UV irradiation at 320 nm for 30 min as recommended by the manufacturer. Immediately prior to microdissection, slides were deparaffinized, rehydrated and stained with Mayers hemalum solution (Merck Millipore, Darmstadt, Germany). Lymphocyte and macrophage exclusive regions in metastized lymph nodes were selected, cut and catapulted into individual PALM® adhesive cap microcentrifuge tubes (Carl Zeiss MicroImaging GmbH, Germany). Microcentrifuge tubes with the areas of interest were transported on ice and RNA was extracted immediately.

### RNA extraction and real-time PCR

Isolation of total RNA was performed with the Absolutely RNA FFPE kit (Agilent Technologies, California, USA), according to the manufacturers’ protocol. Briefly, sections from each archival sample were deparaffinized and incubated overnight with a lysis buffer containing proteinase K and submitted to a series of washes-on-column until elution. Immediately after, 50 ng of RNA were reversed transcribed with Maxima First Strand cDNA Synthesis Kit (Thermo Fisher Scientific, MA, USA) in a total volume of 20 μL, according to the manufacturer’s protocol. Evaluation of FPN1 mRNA levels (Hs00221783_m1) was performed in a Rotor-Gene 6000 instrument (Qiagen, CA, USA) with a TaqMan® Probe-based gene expression assay (Applied Biosystems, CA, USA). Reactions were carried out in triplicate and gene expression levels calculated relative to GUSB mRNA levels (Hs99999908_m1). Mean relative expression was calculated based on the formula ΔCt = Ct target gene—Ct endogenous control gene and fold change on 2^(ΔCt breast tumor samples – ΔCt normal breast samples)^.

### Statistical analysis

Sample distributions were compared using Kruskal-Wallis or Mann-Whitney’s U tests. Pearson’s Chi-Square was used to evaluate the differences between categorical variables. The Spearman’s rank correlation coefficient was used to evaluate the relationship between variables. In figures, experimental errors are shown as one standard error of the mean. Data were analyzed in IBM SPSS Statistics 20.0 software and statistical significance was accepted at *p* < 0.05.

## Results

### Immunolocalization and relative expression of iron-related proteins in breast tissue

**Fig. 1:**
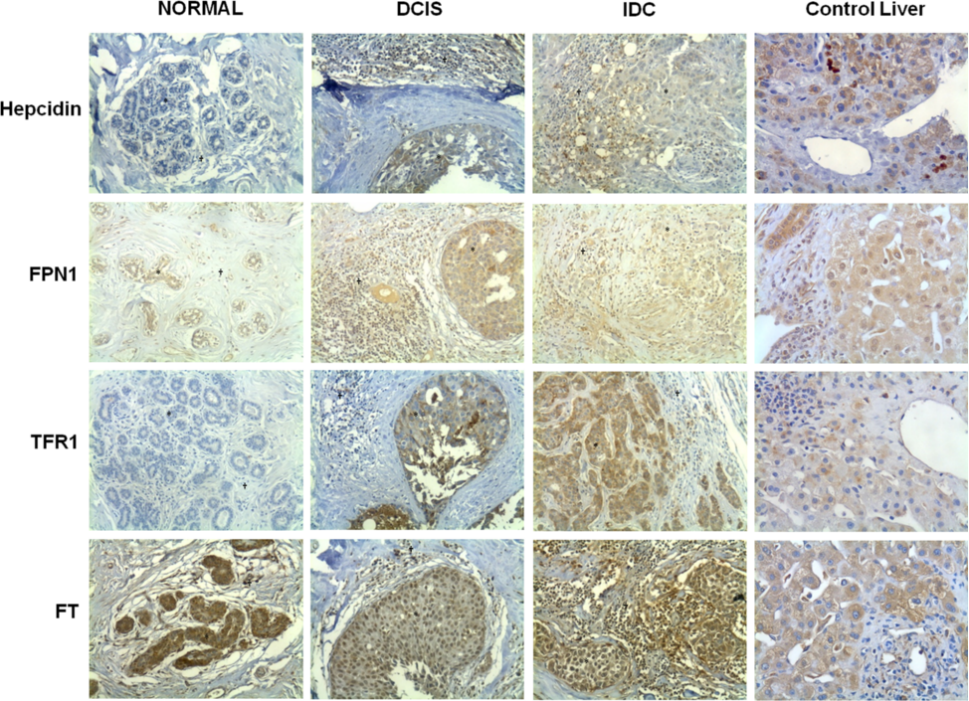
Hepcidin, FPN1, TFR1 and FT expression pattern in breast tissue. Representative images of Hepcidin, FPN1 (Ferroportin 1), TFR1 (Transferrin Receptor 1) and FT (Ferritin) immunostaining in normal breast, DCIS (ductal carcinoma *in situ*) and IDC (invasive ductal carcinoma). A section of human donor liver is also shown as a positive control. Tissue microarrays containing several samples of breast tissue and human donor liver were constructed, sectioned and subjected to immunohistochemistry, as described in materials and methods (Original magnification × 400). * and † are representative of epithelial cells and leukocyte infiltrate, respectively

**Fig. 2:**
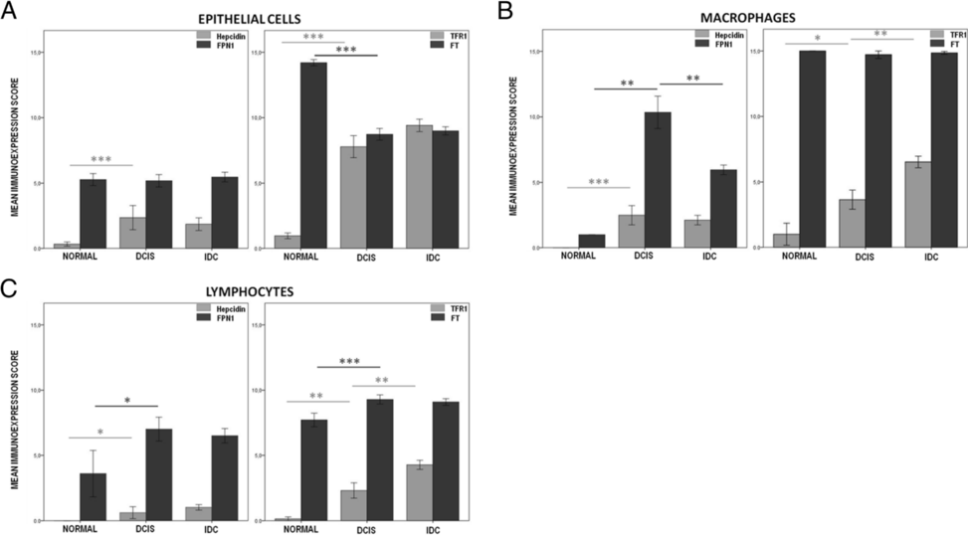
Hepcidin/FPN1 and TFR1/FT phenotype dyads in breast tissue. A semi-quantitative method of assessing the immunoexpression in the TMA sections was applied by multiplying the area and intensity staining scores, as described in materials and methods. The scores ranged from 0 to 15. Graphs show Mean ± SEM. Significant differences are shown for comparison with the precedent group *p < 0.05, **p < 0.01, ***p < 0.001, Mann Whitney’s U test; **a** Immunoexpression score for breast epithelial cells in control normal samples, DCIS (ductal carcinomas *in situ*) and IDC (invasive ductal carcinomas) for hepcidin (*n* = 121), FPN1 (Ferroportin 1, *n* = 113), TFR1 (Transferrin Receptor 1, *n* = 119) and FT (Ferritin, *n* = 119); **b** Immunoexpression score for macrophages in control normal samples, DCIS (ductal carcinomas *in situ*) and IDC (invasive ductal carcinomas) for Hepcidin (*n* = 75), FPN1 (Ferroportin 1, *n* = 62), TFR1 (Transferrin Receptor 1, *n* = 73) and FT (Ferritin, *n* = 91); **c** Immunoexpression score for lymphocytes in control normal samples, DCIS (ductal carcinomas *in situ*) and IDC (invasive ductal carcinomas) for Hepcidin (*n* = 77), FPN1 (Ferroportin 1, *n* = 70), TFR1 (Transferrin Receptor 1, *n* = 73) and FT (Ferritin, *n* = 91)

Immunolocalization of hepcidin, FPN1, TFR1 and FT was assessed in breast tissue samples of normal controls, DCIS and IDC cases. As seen in the representative images illustrated in Fig. 1, different staining patterns were apparent among sample types and, within samples, among the different cell types. Leukocyte infiltrate was much more pronounced in carcinoma than in normal mastectomy samples. Using the semi-quantitative data (described in Methods) obtained exclusively in representative cores of “pure” lesions, we assessed the expression of hepcidin, FPN1, TFR1 and FT in epithelial cells, lymphocytes and macrophage in normal and cancer (DCIS and IDC) breast tissue. The results are illustrated in Fig. 2.

### Hepcidin

Hepcidin expression was restricted to the cytoplasm in all cell types detected. Breast cancer epithelial cells (in DCIS and IDC) presented a significantly higher expression of hepcidin than in control normal samples (*p* < 0.001) (Fig. 2a). The pattern of differential expression was similar for the stromal inflammatory cells analyzed. Breast cancer infiltrating lymphocytes and macrophages also presented significantly higher expression of hepcidin (*p* = 0.002 and *p* < 0.001, respectively) (Fig. 2b,c).

### Ferroportin 1

FPN1 expression in breast epithelial cells was mainly observed in the cytoplasm but, in some cases, also in the cell membrane. In lymphocytes and macrophages it could only be detected in the cytoplasm. Regarding epithelial cells, no significant differences were observed for FPN1 expression between normal samples, DCIS and IDC (Fig. 2a). However, in breast carcinoma samples, lymphocytes and macrophages expressed significantly higher levels of FPN1 than in normal samples (*p* = 0.014 and *p* < 0.001, respectively) (Fig. 2b,c), with FPN1 expression in macrophages being higher in DCIS samples (*p* < 0.01 when compared with IDC samples) (Fig. 2b).

**Fig. 3:**
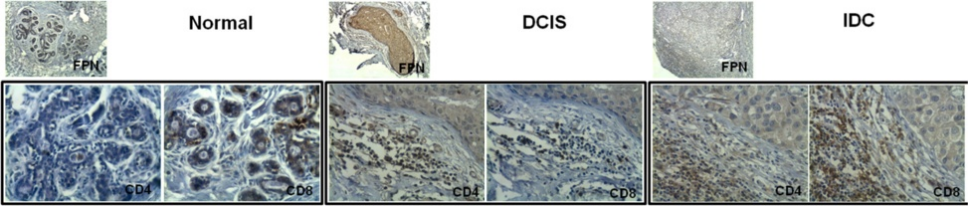
FPN1-expressing leukocytes are composed by a mixture of CD4 and CD8 T-cells. Sections of normal breast tissue, DCIS (ductal carcinoma *in* situ) and IDC (invasive ductal carcinoma) to reveal the presence of CD4 and CD8 T cells, in FPN1-expressing leukocyte infiltrate. For details see Materials and Methods (Original magnification × 100- upper FPN images, ×400- CD4 and CD8 images)

**Fig. 4:**
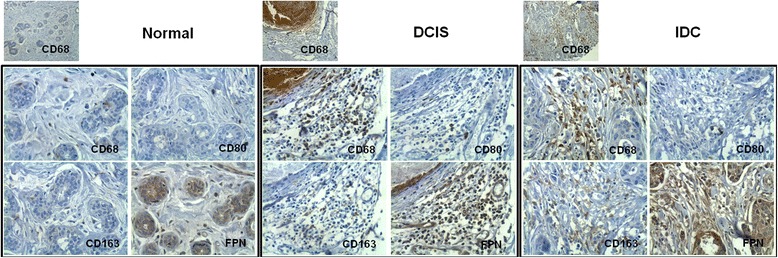
FPN1-expressing leukocytes in carcinomas are predominantly M2-like. Sections of normal breast tissue, DCIS (ductal carcinoma *in* situ) and IDC (invasive ductal carcinoma) to reveal the presence of cells of the macrophage lineage (CD68), and its classical polarization phenotypes, M1-like (CD80) and M2-like (CD163), in FPN1-expressing leukocyte infiltrate. For details see Materials and Methods (Original magnification × 100- upper CD68 images, ×400- squared image series)

Samples with FPN1-expressing T-lymphocytes are composed by a combination of CD4 and CD8 cells (Fig. 3). Tissue section staining with CD68 (macrophage lineage marker), CD80 (M1-like) and CD163 (M2-like) led to the observation that while in normal samples the macrophage population comprises a combination of comparable numbers of cells expressing CD80 and CD163, in breast carcinoma samples this population is predominantly composed of CD163-positive cells, and thus associated with an M2 (alternative) macrophage polarization phenotype (Fig. 4).

### Transferrin receptor 1

TFR1 expression was predominantly detected in the cytoplasm of all the cell types analyzed. Nonetheless, in epithelial cells of some breast carcinoma samples a clear membranar staining was also observed. TFR1 expression was significantly higher in epithelial cells, lymphocytes and macrophages from breast carcinoma samples (*p* < 0.001) in comparison with control normal samples (Fig. 2). Furthermore, TFR1 immunoexpression in infiltrating lymphocytes and macrophages was, as expected, higher in IDC samples (*p* < 0.01) when compared with DCIS (Fig. 2b,c).

### Ferritin

FT expression was predominantly observed in the cytoplasm of epithelial cells, lymphocytes and macrophages. Breast cancer epithelial cells presented a significantly lower expression of FT than normal samples (*p* < 0.001) (Fig. 2a). On the other hand, FT expression in breast cancer infiltrating lymphocytes was significantly higher than in normal samples (*p* < 0.001) (Fig. 2c). No significant differences were found regarding FT in macrophages, given that its expression was consistently high in all the samples analyzed (Fig. 2b). Nuclear FT staining in epithelial cells was also noted. FT staining was also present in tissue stromal fibers of some IDC cases.

**Fig. 5:**
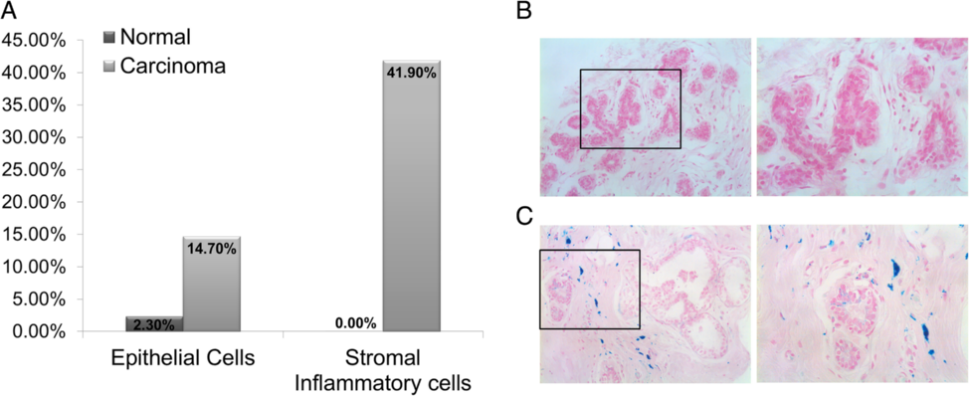
Breast cancer tissue presents a higher accumulation of iron than normal breast. **a** Percentage of normal and breast cancer samples presenting hemosiderin deposits in epithelial and stromal inflammatory cells; **b-c** Representative images of Perls’ iron staining of a normal (**b**) and DCIS (**c**) sample, showing pronounced deposition of hemosiderin in stromal inflammatory cells (arrows) and to a lesser extent in ductal epithelial cells (asterisk) (Original magnification × 200, ×400)

In agreement with these results, suggesting an effective iron ‘reservoir’ in lymphocytes and macrophages, hemosiderin detection through Perls staining demonstrated that a significantly higher proportion of carcinoma cases, when compared with control normal samples, present hemosiderin deposits in stromal inflammatory (*p* = 0.002) and epithelial cells (*p* = 0.033) (Fig. 5).

### Comparative expression of iron-related proteins in pure DCIS lesions and DCIS in IDC

**Fig. 6:**
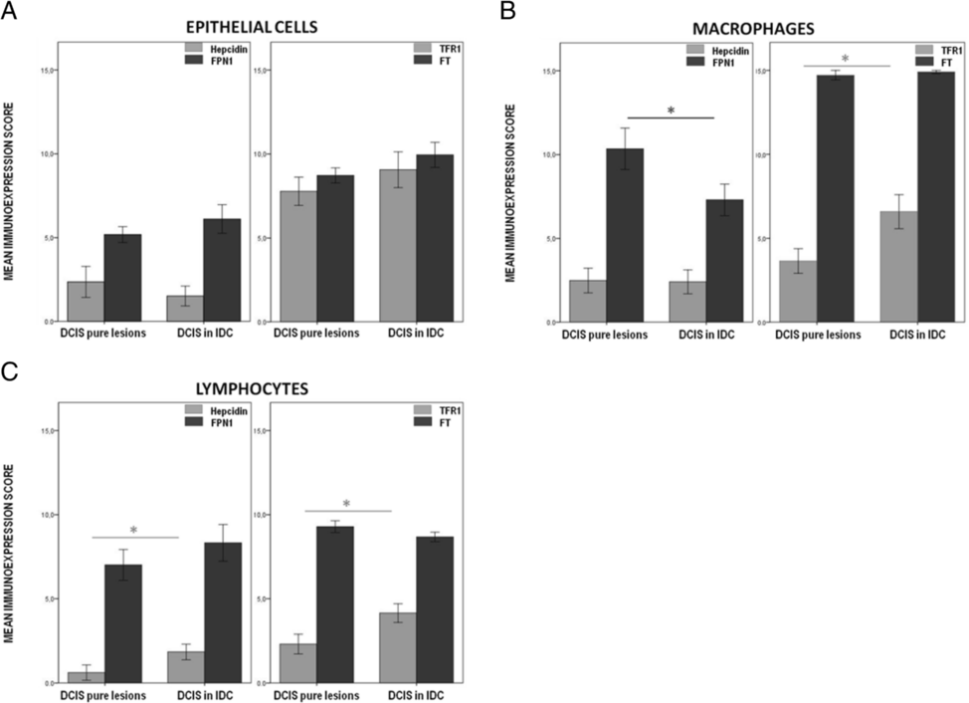
Hepcidin/FPN1 and TFR1/FT phenotype dyads in ductal carcinoma *in situ* lesions (DCIS). A semi-quantitative method of assessing the immunoexpression in the TMA sections was applied by multiplying the area and intensity staining scores, as described in materials and methods. The scores ranged from 0 to 15. Graphs show Mean ± SEM. Significant differences are shown for comparison with the precedent group *p < 0.05, **p < 0.01, ***p < 0.001, Mann Whitney’s U test; **a** Immunoexpression score for breast epithelial cells in DCIS pure lesions and DCIS lesions in IDC for Hepcidin (*n* = 35), FPN1 (Ferroportin 1, *n* = 35), TFR1 (Transferrin Receptor 1, *n* = 32) and FT (Ferritin, *n* = 36); **b** Immunoexpression score for macrophages in DCIS pure lesions and DCIS lesions in IDC for Hepcidin (*n* = 28), FPN1 (Ferroportin 1, *n* = 27), TFR1 (Transferrin Receptor 1, *n* = 30) and FT (Ferritin, *n* = 33); **c** Immunoexpression score for lymphocytes in DCIS pure lesions and DCIS lesions in IDC for Hepcidin (*n* = 26), FPN1 (Ferroportin 1, *n* = 31), TFR1 (Transferrin Receptor 1, *n* = 30) and FT (Ferritin, *n* = 33)

We demonstrated that the deregulated expression of iron-related proteins in breast cancer is not restricted to the tumor cells, but extends to the lymphocytes and macrophages in the tumor microenvironment. Given the fact that FPN1 expression in macrophages is particularly high in pre-invasive stages (DCIS), we sought to verify if these iron-related phenotypes were specific of pure DCIS or if they could also be observed in DCIS lesions adjacent to invasive ductal carcinomas (DCIS in IDC). The results are illustrated in Fig. 6.

Epithelial cells from DCIS pure lesions or from DCIS in IDC did not exhibit significant differences regarding the expression of the previously assessed iron-related proteins (Fig. 6a). Major differences were found, however, for tumor-associated lymphocytes and macrophages. Lymphocytes had a significantly higher expression of hepcidin (*p* = 0.030) and TFR1 (*p* = 0.011) in DCIS in IDC (Fig. 6c), while macrophages from DCIS pure lesions exhibited a higher expression of FPN1 than DCIS in IDC (*p* = 0.036) (Fig. 6b).

### Imaging flow cytometry

**Fig. 7:**
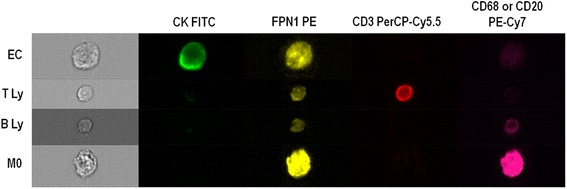
Representative images of the FPN1 analysis by Imaging Flow Cytometry in breast cancer core biopsies. Epithelial cells (EC) are stained by an anti-cytokeratin (CK) FITC. T lymphocytes (T Ly) were identifiable by CD3 PerCP-Cy5.5, B lymphocytes (B Ly) by CD20 PE-Cy7 and macrophages (M0) by CD68 PE-Cy7 (Original magnification × 400)

In order to confirm the expression of FPN1 in epithelial cells, lymphocytes and macrophages from breast carcinoma samples and further explore its cellular distribution we resourced to imaging flow cytometry to relatively quantify it and determine its localization. For that purpose, we used OCT-frozen tissue from 6 patients with invasive ductal carcinomas, and a panel of antibodies to identify epithelial cells, T and B lymphocytes and macrophages (described in Methods). Furthermore, a mask to identify specifically the cell membrane and cytoplasm was built in IDEAS v6.0.348 software to evaluate FPN1 expression in each cell compartment. The ratio between the median FPN1 intensity in the cytoplasm and membrane was calculated as a putative surrogate for the iron export capacity of the cell. Representative images are shown in Fig. 7 and the results are summarized in Table 2. FPN1 expression could be detected by Imaging Flow Cytometry in epithelial cells, T lymphocytes, B lymphocytes and macrophages. Macrophages in breast cancer tissue presented the highest median fluorescence intensity of the cell types considered, as a confirmation of the results presented in Fig. 2b. The ratio between the median FPN1 intensity in the cytoplasm and membrane allowed us to notice that FPN1 expression was higher in the cytoplasm of all the cell types considered, when compared with the membrane.

**Table 2:** FPN1 median expression in IDC samples assessed by Imaging Flow Cytometry

Cell type	Mean number of cells on focus ± SEM	Total cell FPN1 PE MFI ± SEM	Cytoplasm FPN1 PE MFI ± SEM	Membrane FPN1 PE MFI ± SEM	Ratio cyt/memb FPN1 PE MFI ± SEM
EC	3935 ± 605	32.23 ± 4.26	46.08 ± 5.98	18.77 ± 2.32	2.47 ± 0.22
T Ly	53 ± 13	11.99 ± 0.63	15.7 ± 1.14	7.22 ± 0.22	2.31 ± 0.20
B LY	11 ± 7	15.09 ± 2.63	18.76 ± 2.04	9.41 ± 0.87	2.03 ± 0.03
M0	154 ± 38	69.82 ± 9.26	105.55 ± 23.82	53.11 ± 7.53	2.00 ± 0.001

*Abbreviations*: *FPN1* Ferroportin 1, *IDC* Invasive Ductal Carcinoma, *MFI* Median Fluorescence Intensity, *SEM* Standard Error of the mean, *Cyt* Cytoplasm, *Memb* Membrane, *EC* Epithelial Cells, *Ly* Lymphocytes, *M0* Macrophages

### Lymph nodes

**Fig. 8:**
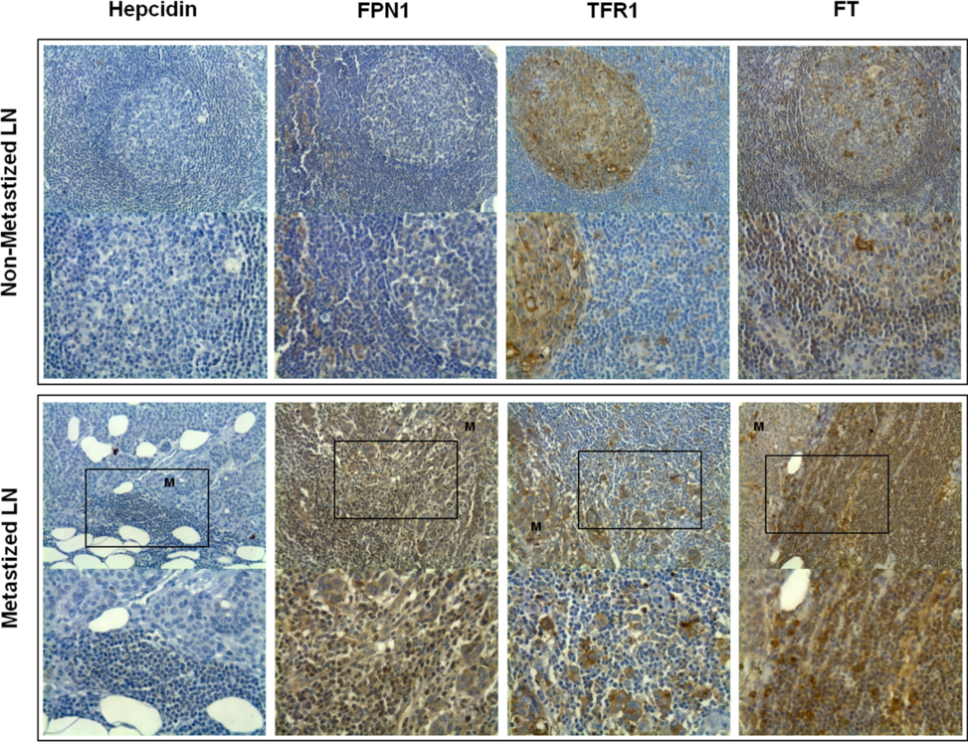
Representative images of Hepcidin, FPN1, TFR1 and FT immunostaining in non-metastized and metastized lymph nodes. Archived lymph nodes from cases with previously analyzed primary invasive ductal carcinomas were sectioned and subjected to immunohistochemistry, as described in materials and methods. Boxes indicate lymph node areas near metastasis. Note prominent germinal centers in non-metastized lymph nodes (×400 original magnification figures are shown below its × 200 correspondent figures). M, metastasis; LN, lymph node

Considering that the expression of iron-related proteins in lymphocytes and macrophages varied in different tumor microenvironments, we extended the observation to metastized and non-metastized lymph nodes from the original cohort of patients, whose primary tumors had been previously analyzed. Hepcidin, FPN1, TFR1 and FT immunoexpression were assessed in 14 non-metastized and 12 metastized lymph-nodes (Fig. 8) and semi-quantitatively scored (Table 3).

**Table 3:** Analysis of iron-related proteins expression in the LN of patients with IDC

		Non-metastized LN	Metastized LN	
	Cell type	Mean ± SEM	Mean ± SEM	*p*
Hepcidin	B cell areas	0.08 ± 0.08	0.62 ± 0.14	*0.005*
	T cell areas	0.15 ± 0.10	0.62 ± 0.14	*0.018*
	M0	2.00 ± 0.00	1.92 ± 0.08	ns
FPN1	B cell areas	1.38 ± 0.14	2.23 ± 0.17	*0.002*
	T cell areas	1.38 ± 0.14	2.23 ± 0.17	*0.002*
	M0	1.92 ± 0.08	2.23 ± 1.17	ns
TFR1	B cell areas	1.92 ± 1.18	1.08 ± 0.27	*0.026*
	T cell areas	1.00 ± 0.11	1.08 ± 0.08	ns
	M0	2.00 ± 0.11	2.31 ± 0.13	ns
FT	B cell areas	1.77 ± 0.12	2.77 ± 0.12	*<0.001*
	T cell areas	1.54 ± 0.14	2.62 ± 0.14	*<0.001*
	M0	3.00 ± 0.00	3.00 ± 0.00	ns

*Abbreviations*: *LN* Lymph Node, *IDC* Invasive Ductal Carcinoma, *SEM* Standard Error of the Mean, *M0* Macrophages, *FPN1* Ferroportin 1, *TFR1* Transferrin Receptor 1, *FT* Ferritin, *NS* Not Statistically Significant

Hepcidin immunoexpression in lymph nodes was mostly restricted to macrophages and scarcely observed in lymphocytes. Metastized lymph nodes, however, presented a significantly higher immunoexpression of hepcidin in lymphocytes (B cell areas: *p* = 0.005; T cell areas: *p* = 0.018), than in non-metastized lymph-nodes.

FPN1 was uniformly expressed in all the cell types. In lymph nodes with sinus histiocytosis, lymphocytes and macrophages had a tendency to lower FPN1 expression. Remarkably, lymphocytes in metastized lymph nodes expressed 1.80-fold more FPN1 than in non-metastized ones, particularly in areas adjacent to the metastasis (*p* = 0.002). Expression assessment at the mRNA level confirmed a 1.48-fold increase in FPN1 expression in leukocyte areas of metastized lymph nodes, comparing with non-metastized lymph nodes (*p* = 0.057).

TFR1 was mostly expressed in macrophages and germinal center cells, particularly in non-metastized lymph nodes (*p* = 0.026). TFR1 was seldom expressed in T-cell areas, with no significant differences observed between metastized and non-metastized lymph nodes.

While the immunoexpression pattern of FT was similar to FPN1, a significantly higher expression of FT was observed for lymphocytes (*p* < 0.001), noted particularly near metastasis areas.

### Clinicopathological data

The expression of iron-related proteins was finally correlated with clinicobiological markers of breast cancer behavior, specifically hormone receptor and HER2 status. Results of mean FPN1 expression values in epithelial cells and macrophages in DCIS and IDC lesions are shown in Table 4 in relation to the ER, PR and HER2 status. FPN1 expression in IDC lesions was significantly higher in ER negative (*p* = 0.018) and in HER2 positive cases (*p* = 0.001) in epithelial cells, whereas in DCIS lesions FPN1 expression was only associated with negative ER status in macrophages (*p* = 0.033). No associations were found between FPN1 expression and PR status. Regarding TFR1 expression, a significantly higher expression was seen in macrophages of negative PR DCIS cases (*n* = 15; *p* = 0.039) and in lymphocytes and macrophages of HER2 positive IDC cases (Ly: *n* = 79; *p* = 0.028; M0: *n* = 79; *p* = 0.003). A higher expression of FT in lymphocytes was observed in negative PR DCIS cases (*n* = 15; *p* = 0.029). All other comparisons were not statistically significant.

**Table 4:** FPN1 expression in epithelial cells and macrophages in carcinomas according to clinicopathological variables

	DCIS (Mean ± SEM)				IDC (Mean ± SEM)			
Clinicopathological variable	EC	*Significance level.*	M0	*Significance level.*	EC	*Significance level.*	M0	*Significance level.*
ER status	*n* = 22	*ns*	*n* = 14	*p = 0.033*	*n* = 103	*p = 0.018*	*n* = 73	*ns*
ER-	6.29 ± 0.65		13.04 ± 0.87		7.47 ± 0.88		7.17 ± 0.95	
ER+	4.51 ± 0.43		7.75 ± 2.14		5.18 ± 0.31		6.01 ± 0.34	
PR status	*n* = 22	*ns*	*n* = 14	*ns*	*n* = 103	*ns*	*n* = 73	*ns*
PR-	6.01 ± 0.61		11.59 ± 1.64		6.47 ± 0.65		7.25 ± 0.77	
PR+	4.48 ± 0.46		9.30 ± 1.80		5.40 ± 0.36		5.83 ± 0.35	
HER2 status	*n* = 22	*ns*	*n* = 14	*ns*	*n* = 101	*p = 0.001*	*n* = 73	*ns*
HER2-	5.76 ± 0.63		9.00 ± 2.45		5.21 ± 0.35		6.02 ± 0.38	
HER2+	4.98 ± 0.57		11.76 ± 1.34		7.47 ± 0.64		6.89 ± 0.74	

*Abbreviations*: *DCIS* Ductal Carcinoma *In Situ*, *IDC* Invasive Ductal Carcinoma, *ER* Estrogen Receptor, *PR* Progesterone Receptor, *HER2* Human Epidermal growth factor Receptor 2, *EC* Epithelial Cells, *Ly* Lymphocytes, *M0* Macrophages, *FPN1* Ferroportin 1, *TFR1* Transferrin Receptor 1, *FT* Ferritin, *ns* Not Statistically Significant, *SEM* Standard Error of the Mean

We next analyzed the expression of these iron-related proteins in relation to local and metastatic tumor growth in invasive tumors (Table 5). Tumor size was positively correlated with TFR1 expression in all the cell types considered (EC: *p* = 0.027; *r* = 0.226; Ly: *p* = 0.041; *r* = 0.235; M0: *p* = 0.017; *r* = 0.274).

**Table 5:** Correlation table between tumor size and TFR1 expression

	TFR1 (Mean ± SEM)					
Clinicopathological variable	EC	Corr. Coeff. Sig	Ly	Corr. Coeff. Sig	M0	Corr. Coeff. Sig
Tumor size	*n* = 96	*p = 0.027*	*n* = 76	*p = 0.041*	*n* = 76	*p = 0.017*
T1	6.69 ± 0.56		3.37 ± 0.31		5.39 ± 0.47	
T2	7.83 ± 0.93		4.99 ± 0.57		6.94 ± 0.63	
> T3	9.00 ± 4.83		3.47 ± 0.65		7.00 ± 1.34	

*Abbreviations*: *EC* Epithelial Cells, *Ly* Lymphocytes, *M0* Macrophages, *TFR1* Transferrin Receptor 1, *SEM* Standard Error of the Mean

Lymph node involvement was not associated with the expression of these iron-related proteins in the primary tumor tissue. Of notice, the peripheral blood leukocyte count at the time of diagnosis was also correlated with the expression of TFR1 and FPN1 in primary tumor’s lymphocytes (TFR1: *p* = 0.001; *r* = 0.355; FPN1: *p* = 0.017; *r* = 0.274) and macrophages (TFR1: *p* = 0.002; *r* = 0.367; FPN1: *p* = 0.034; *r* = 0.244).

## Discussion

We would like to start this discussion by placing the present results in the growing interest on the tissue microenvironment contribution for malignancy [16, 29, 30]. Thus far, most of this interest has focused on cytokines and immune response to putative tumor antigens [31, 32]. Recently, however, interest has grown in the interaction of migrating cells to the tissue microenvironment, namely macrophages, neutrophils and certain lymphocyte subsets [14, 33, 34].

Cells migrating to a tumor microenvironment must benefit in general from the tumor associated development of new vessels [35]. Angiogenesis is thought to provide nutritional advantage to the transformed malignant cell. Yet, very few studies have focused on an obvious nutrient associated with cell division, such as iron. The present study sought, to a certain extent compensate for that scanty interest.

Thus, we approached the question of the iron homeostasis deregulation in breast cancer by analyzing the specific iron-related phenotypes of different cell types present in the tumor tissue namely epithelial cells, lymphocytes and macrophages, and correlating the iron-related phenotypes with clinicopathological markers of disease prognosis. The analysis of iron-related phenotypes in breast ductal carcinoma epithelial cells confirmed previous observations that they display a phenotype of relative iron deficiency, characterized by a marked increase in TFR1 expression (for review see [36]) and downregulation of FT [21]. Although we observed an increase in hepcidin expression in breast cancer tissue, as previously described, we were not able to demonstrate a concomitant decrease in FPN1 expression in breast cancer epithelial cells, compared with normal epithelial cells [12, 13, 37]. Although not analyzed in our study, results from Wang et al. suggest that the ‘iron-deficient’ phenotype of breast cancer cells may be driven by the increased expression of the iron-regulatory protein (IRP) 2 [38]. The discrepancy found with previous reports regarding FPN1 might be due to the inclusion, in those studies, of different breast cancer types besides ductal carcinomas or by the assessment of FPN1 at the transcriptional level instead of the protein level, or still due to our limited number of samples. Also, we cannot exclude the influence of other regulatory mechanisms on FPN1 expression, other than hepcidin-mediated ferroportin 1 downregulation at the post-translational level, such as epigenetic mechanisms, deregulation of Nrf2 and MZF-1 expression [37] or a HIF-2α dependent pathway [39].

The analysis of iron-related phenotypes in stromal inflammatory cells revealed that infiltrating macrophages and lymphocytes display an “iron-donor” phenotype with increased expression of both FPN1 and FT concomitant with an activation profile reflected by a higher expression of hepcidin and TFR1. The increased FPN1 expression was particularly evident in macrophages of DCIS lesions (see Figs. 2 and 6), but was also clear in lymphocytes, not only in the primary tumor site but also in metastized lymph nodes (see Figs. 2c and 8 and Table 3). The simultaneous overexpression of TFR1 and FT, and hepcidin and FPN1 in lymphocytes and macrophages questions the established principles of iron-related proteins’ regulation at the post-transcriptional and –translational levels. Considering the role of IRPs 1 and 2 in a situation of high iron levels, as expected in a breast cancer setting, IRP binding to 5′- untranslated region (UTR) of FT and lack of stabilization at the 3′-UTR of TFR1 would lead to an increased FT translation, with a concomitant decrease in TFR1. Unexpectedly, this was not observed, Although TFR1 is classically viewed, in this context, for its role in iron acquisition and malignant cell nutrition, there is evidence showing an alternative role for TFR1 in the activation of T cells, independently of iron-uptake [36, 40, 41]. Furthermore, we cannot disregard the fact that FT detection was achieved with a polyclonal antibody, not discriminating the heavy and light subunits, which could be argued as not reflecting iron accumulation in these cells. However, the fact that we also demonstrated that over 40 % of DCIS and IDC samples present stromal inflammatory cells with hemosiderin deposits supports our hypothesis that these cells may constitute an effective iron reservoir potentially contributing to tumor nutrition. The observed concurrent increased expression of hepcidin and FPN1 in lymphocytes and macrophages of breast ductal carcinomas may also argue in favor of such a nutritional role. Studies from others have also demonstrated a similar hepcidin-independent mechanism of iron export, reflecting a role for heme. They showed that heme derived from erythrophagocytosis can stimulate FPN1 transcription in primary cultures of bone marrow derived macrophages and that hepcidin was not able to block iron-heme export during erythrocyte-iron recycling by macrophages [42, 43].

Markers of iron deregulation in stromal cells were also found here significantly associated with other clinicopathological markers of poor prognosis, namely hormone receptor status negativity and tumor size (Tables 4 and 5). Several studies had already focused on the establishment of associations between the immune profile of the infiltrating leukocytes in the tumor and established clinicopathological variables of breast cancer outcome [44–46] but very few have approached the association with iron-related proteins. Interestingly, Britten et al. had already demonstrated that spleens from patients with Hodgink’s disease presented a higher expression of ferritin in macrophages, particularly around tumor nodules [47]. The present results point to the fact that deregulation of iron metabolism occurs not only at the primary tumor microenvironment, but also in preferential metastatic niches. To our knowledge, only Jezequel and coworkers have previously described the expression of an iron-related protein (ferritin light-chain, FTL) in stromal cells as a prognostic marker in node-negative breast cancer patients [22]. In the present study, and for the first time, an association between the expression of iron-related proteins in lymphocytes and macrophages and negative hormone receptor status in DCIS and tumor size was demonstrated*.* Moreover, neither the expression of FPN1 and FT in axillary lymph nodes nor the association of FPN1 and FT overexpression in lymphocytes with the presence of breast cancer metastasis has been previously described. In summary, these significant associations observed for lymphocytes and macrophages reinforce a reason for interest in their contribution for the tumor microenvironment.

This study raises some new questions that deserve to be analyzed. The first one is how stromal cells acquire their “iron donor” phenotype. Are they responding locally to signals derived from cancer cells? Are they mobilized from the peripheral blood with this phenotype? Further studies should be performed to clarify this question. One may consider however that, at the systemic level, macrophages and lymphocytes constitute important iron storage compartments involved, respectively, in the recycling of iron from senescent erythrocytes and in the uptake of non-transferrin-bound iron [48–50]. Based on the original hypothesis of de Sousa of the immune system in the surveillance of the potential iron toxicity associated with red blood cell circulation and more recently extended by Pinto et al. work in 2014 [49, 51], this circulating compartment might, indeed, be responsible for delivering iron locally in situations of increased blood flow, such as in tumor-derived angiogenesis, and hence contribute to tumor sustained growth through iron nutrition [52]. This notion is further supported by our present data showing a highly significant correlation between the number of circulating leukocytes at the time of diagnosis and the high expression of FPN1 and TFR1 in breast cancer infiltrating lymphocytes and macrophages.

## Conclusion

In summary, the results presented here confirm that the deregulation of iron metabolism is an aspect common to several cellular types of breast cancer tissue, and not restricted to epithelial cells. Moreover, this deregulation of iron-related proteins in infiltrating lymphocytes and macrophages add evidence to the view that stromal cell responses in the breast microenvironment may contribute critically to tumor progression [53, 54].

With the present cross-sectional approach it was not possible to establish a real timeline for breast cancer development and progression. Moreover, the fact that this is an anonymous cross-sectional study without access to follow-up data, has limited the comprehension of the value of iron-related alterations in infiltrating lymphocytes and macrophages of the breast tumor microenvironment. Subsequent prospective studies monitoring the expression of these iron-related proteins in patients are still needed to validate the significance of local iron-profiles as relevant markers of breast disease progression. Furthermore, the analysis of IRP expression in specific breast tissue cell types may provide additional insight into the regulation of iron homeostasis in breast cancer. Future in vitro studies should be designed in order to confirm not only the capacity of lymphocytes and macrophages to donate iron to breast epithelial cells but also to explore how malignant cells could influence their environment in order to acquire iron beyond stimulating angiogenesis.

### Ethics approval

This project was approved by the following ethical boards: Centro Hospitalar do Porto Research Ethics Health Committee (references 228-CES; 203-CES) and by Centro Hospitalar do Porto Department of Education, Development and Research (references 152-DEFI; 135-DEFI). Informed consent from the patients whose tissue was archived was not required as per Ethics Board guidelines. Patient informed consent for the use of breast core biopsies for flow cytometry studies was obtained.

## Abbreviations

BSA: bovine serum albumin
DCIS: ductal carcinoma in situ
EC: epithelial cell
ER: estrogen receptor
FFPE: formalin-fixed paraffin-embedded
FPN1: ferroportin 1
FT: ferritin
H&E: hematoxylin and eosin
HER-2: human epidermal growth factor 2
IDC: invasive ductal carcinoma
Ly: lymphocyte
M0: macrophage
OCT: optimum cutting temperature
PR: progesterone receptor
TFR1: transferrin receptor 1
TMA: tissue microarray
UTR: unstranslated region
WBC: white blood cell
